# Feedback preferences of patients, professionals and health insurers in integrated head and neck cancer care

**DOI:** 10.1111/hex.12567

**Published:** 2017-06-15

**Authors:** Lydia F. J. van Overveld, Robert P. Takes, Thomas W. Vijn, Jozé C. C. Braspenning, Jan P. de Boer, John J. A. Brouns, Rolf J. Bun, Boukje A. C. van Dijk, Judith A. W. F. Dortmans, Emilie A. C. Dronkers, Robert J. J. van Es, Frank J. P. Hoebers, Arvid Kropveld, Johannes A. Langendijk, Ton P. M. Langeveld, Sjoukje F. Oosting, Hendrik P. Verschuur, Jan G. A. M. de Visscher, Stijn van Weert, Matthias A. W. Merkx, Ludi E. Smeele, Rosella P. M. G. Hermens

**Affiliations:** ^1^ Radboud University Medical Center Radboud Institute for Health Sciences Scientific Center for Quality of Healthcare Nijmegen The Netherlands; ^2^ Department of Otolaryngology, Head and Neck surgery Radboud University Medical Center Radboud Institute for Health Sciences Nijmegen The Netherlands; ^3^ The Netherlands Federation of University Medical Centres, NFU Utrecht The Netherlands; ^4^ Department of Medical Oncology Antoni van Leeuwenhoek Nederlands Kanker Instituut Amsterdam The Netherlands; ^5^ Department of Oral and Maxillofacial Surgery Rijnstate Hospital Arnhem The Netherlands; ^6^ Department of Oral and Maxillofacial Surgery Medical Centre Alkmaar Alkmaar The Netherlands; ^7^ Department of Research Comprehensive Cancer Organization the Netherlands (IKNL) Utrecht The Netherlands; ^8^ Department of Epidemiology University of Groningen University Medical Centre Groningen Groningen The Netherlands; ^9^ Department of Radiation Oncology Medisch Spectrum Twente Enschede The Netherlands; ^10^ Department of Otorhinolaryngology Head and Neck surgery Erasmus MC Cancer Institute Rotterdam The Netherlands; ^11^ Department of Head and Neck Surgical Oncology UMC Utrecht Cancer Center Utrecht The Netherlands; ^12^ Department of Radiation Oncology (MAASTRO) GROW – School for Oncology and Developmental Biology Maastricht University Medical Centre Maastricht The Netherlands; ^13^ Department of Otolaryngology, Head and Neck surgery Elisabeth‐TweeSteden ziekenhuis Tilburg Tilburg The Netherlands; ^14^ Department of Radiation Oncology University of Groningen University Medical Centre Groningen Groningen The Netherlands; ^15^ Department of Otorhinolaryngology, Head and Neck surgery Leiden University Medical Centre Leiden The Netherlands; ^16^ Department of Medical Oncology University of Groningen University Medical Centre Groningen Groningen The Netherlands; ^17^ Department of Otolaryngology Head and Neck surgery MC Haaglanden‐Bronovo The Hague The Netherlands; ^18^ Department of Oral and Maxillofacial Surgery Medical Centre Leeuwarden Leeuwarden The Netherlands; ^19^ Department of Otolaryngology, Head and Neck surgery VU University Medical Centre Amsterdam The Netherlands; ^20^ Department of Oral and Maxillofacial Surgery Radboud university Medical Centre Radboud Institute for Health Sciences Nijmegen The Netherlands; ^21^ Department of Head and Neck Surgery and Oncology Antoni van Leeuwenhoek Nederlands Kanker Instituut Amsterdam The Netherlands; ^22^ Department of Oral and Maxillofacial Surgery Academisch Medisch Centrum Amsterdam Zuid‐Oost The Netherlands

**Keywords:** audit and feedback, feedback preferences, head and neck cancer, health care quality improvement, integrated health care, quality indicators

## Abstract

**Background:**

Audit and feedback on professional practice and health care outcomes are the most often used interventions to change behaviour of professionals and improve quality of health care. However, limited information is available regarding preferred feedback for patients, professionals and health insurers.

**Objective:**

Investigate the (differences in) preferences of receiving feedback between stakeholders, using the Dutch Head and Neck Audit as an example.

**Methods:**

A total of 37 patients, medical specialists, allied health professionals and health insurers were interviewed using semi‐structured interviews. Questions focussed on: “Why,” “On what aspects” and “How” do you prefer to receive feedback on professional practice and health care outcomes?

**Results:**

All stakeholders mentioned that feedback can improve health care by creating awareness, enabling self‐reflection and reflection on peers or colleagues, and by benchmarking to others. Patients prefer feedback on the actual professional practice that matches the health care received, whereas medical specialists and health insurers are interested mainly in health care outcomes. All stakeholders largely prefer a bar graph. Patients prefer a pie chart for patient‐reported outcomes and experiences, while Kaplan‐Meier survival curves are preferred by medical specialists. Feedback should be simple with firstly an overview, and 1‐4 times a year sent by e‐mail. Finally, patients and health professionals are cautious with regard to transparency of audit data.

**Conclusions:**

This exploratory study shows how feedback preferences differ between stakeholders. Therefore, tailored reports are recommended. Using this information, effects of audit and feedback can be improved by adapting the feedback format and contents to the preferences of stakeholders.

## 1 INTRODUCTION

Much effort has been devoted to improve professional practice and outcomes in health care during the past decades, unfortunately with varying effects. A widely used strategy to improve health care is “audit and feedback”,^1^, ^2^ defined as any summary of clinical performance of health care over a specified period of time, given in a written, electronic or verbal format, offering professionals performance information and motivation to improve.^3^

One of the methods to derive the information for audit and feedback is using quality indicators.^4^, ^5^ Quality indicators are aimed at detecting suboptimal care either in structure or process (eg, the percentage of patients discussed in multidisciplinary team meetings), or outcomes (eg, patient‐reported outcomes [PROs] and experiences [PREs]). They can be used as a tool to guide the process of quality improvement in health care.^6^

Although positive effects of audit and feedback have been reported, namely decreased duration of hospital stay^7^ and decreased mortality rates,^8^ this improvement strategy has not been found to be consistently effective.^2^, ^9^, ^10^, ^11^, ^12^ So far, research has focussed on increasing the effectiveness of feedback, for example by including a worksheet in the feedback to facilitate goal setting^13^ and timing of audit and feedback.^3^, ^14^, ^15^ Audit and feedback researchers have recommended a shift towards comparative effectiveness studies, evaluating how and when audit and feedback components will work, rather than its overall effectiveness.^16^

The format of feedback may significantly affect the interpretation of data.^17^, ^18^, ^19^ However, there is only limited information available regarding formats of feedback, for example on how to summarize and display results of outcome measures in the best way.^20^, ^21^, ^22^ Furthermore, implementation of audit and feedback is likely to be more effective when feedback messages can influence barriers to change behaviour. These barriers appear to differ across individuals.^23^ In addition, most audit and feedback interventions use written or graphical feedback in one uniform format for all recipients.^7^ This will surely not meet the preferences of all recipients, and effects will be low if recipients do not understand the feedback. In developing feedback formats, it is therefore necessary to involve all stakeholders receiving feedback, so as to guarantee that the presentation of feedback meets their preferences.^20^, ^24^

In health care systems worldwide, various stakeholders use feedback on quality indicators for different purposes, such as: (i) patients, who are the recipients of health care and for whom feedback on PROs and PREs can be used to improve and monitor their own or others’ health and health care pathways; (ii) medical specialists, who deliver health care and for whom the feedback on their own delivered care may improve health care; (iii) allied health professionals, including nurses, who have a similar role as medical specialists, although restricted to allied health care; and (iv) health insurers, who search for quality information suitable to create differences in quality of care levels as a basis for their contracting. We hypothesize that by adapting feedback to the preferences of these different stakeholders, they will better respond to the information delivered, and more improvement in effects of audit and feedback could be possible.

In this exploratory study, we aim to investigate the preferences of various stakeholders on receiving feedback, with the Dutch Head and Neck Audit (DHNA) as an example. Head and neck cancers (HNCs) are heterogeneous both biologically as well as in clinical behaviour, and they grow relatively fast in an anatomically and functionally complex area.^25^, ^26^ Patients often have problems with speech, swallowing and physical disfiguration due to treatment,^27^, ^28^ requiring the collaboration of both medical specialists and allied health professionals. Therefore, high‐quality integrated care for patients with this type of tumour is needed.^29^, ^30^ The DHNA uses quality indicators to measure the quality of integrated care for patients with HNC within 14 Dutch hospitals.^31^ By investigating the preferences on feedback of all four stakeholders in the DHNA (medical specialists, allied health care workers, patients and health insurers), including “Why,” “On what aspects” and “How” do you prefer to receive feedback on professional practice and health care outcomes, this study can provide useful tools to potentially improve quality of care by adapting the feedback format and contents to stakeholders’ preferences. This can serve as an example for other integrated oncologic care pathways where audit and feedback will be used or, unfortunately, is still less effective.

## 2 METHODS

### 2.1 Study design

In this exploratory, qualitative study the first author conducted semi‐structured interviews with four stakeholders to investigate preferences on feedback using the “consolidated criteria for reporting qualitative research” checklist (COREQ).^32^ Interviews were transcribed verbatim and qualitatively analysed by the first and third author.

### 2.2 Setting

Approximately, 3000 patients are diagnosed yearly with HNC in the Netherlands.^33^ HNC care is centralized in 14 hospitals: eight Head and Neck Oncology Centres (HNOCs) and six affiliated centres. The affiliated centres have committed themselves to using the same treatment protocols as the related HNOC. The various medical specialists and allied health care professionals involved in HNC care are united in two national foundations: one for medical specialists (NWHHT) and one for allied health professionals (PWHHT). Previously, there were two Dutch patient associations: “Stichting Klankbord” and “NSVG”. The former represented all patients with HNC, the latter only laryngectomized patients. Currently, they collaborate in one Dutch patient association called “Patiëntenvereniging Hoofd‐Hals”. In the Netherlands, there are four major health insurers as well as several smaller companies. In 2014, a quality registration was set up to measure the quality of integrated HNC care, using quality indicators selected by the four stakeholders.^31^

### 2.3 Participants

Four different groups of stakeholders were interviewed about their preferences. Research shows that 13‐15 interviewees are usually sufficient to reach data saturation (the point at which no new information is mentioned in interviews)^34^. Therefore, at least 13 persons were invited for each stakeholder group. However, only the four major health insurers were invited.

A patient panel (including the chairmen of both patient associations) that participated in a previous study was asked by e‐mail to participate again (van Overveld, 2016, unpublished). A letter with additional information about the research methods and an informed consent form were handed over to the patients at a meeting prior to the interview. The location for the personal appointment was either at their home, their work or at the hospital. Medical specialists and allied health professionals and nurses, belonging to the national foundations, were invited to participate in an interview, either by telephone or in person. We aimed to interview at least one professional of each profession (radiation oncologist, medical oncologist, oral and maxillofacial surgeon, otorhinolaryngologist, speech therapist, physiotherapist, dietician, oral hygienist and nursing consultant) involved in HNC care. We contacted the four major health insurers by e‐mail, to ask whether they would be willing to participate in an interview, either by telephone or in person. Persons approached were specialized in health care purchasing policy, innovation and advice or innovation and quality. Prior to an interview by telephone or a meeting, the professionals, patients and health insurers received a document with examples of the type of graphs to be discussed (see Result section Table 6, first column). In this article, the term “professionals” will be used when referring to medical specialists together with allied health professionals, and “allied health professionals” refers to both allied health professionals and nurses.

### 2.4 Data collection

Each interview took approximately 20‐30 minutes and was audio‐recorded. Moreover, all patients signed informed consent forms, while each interviewee received the same questions. Questions focussed on three topics: (i) “Why do you prefer to receive feedback on professional practice and health care outcomes?”, for example reasons for feedback at an individual level, hospital level and national level for indicators on outcome, process and structure; (ii) “On what aspects would you prefer to receive feedback regarding professional practice and health care outcomes?”, for example interest in specific indicators; (iii) “How do you prefer to receive feedback on professional practice and health care outcomes?”, for example frequency, timing, report form, type of graph preferred and transparency, for example whether patients prefer to receive national average scores on PROs and PREs and whether results of quality of care in hospitals can become public. In addition, the interviews with patients were focussed particularly on the PROs and PREs with regard to questioning health care outcomes, because patients had a better understanding of the feedback on these questions compared with feedback on, for example, survival. Questions for the health insurers focussed merely on the goal of feedback, because they will use feedback in a different way compared with patients and health professionals. Different graph types were selected from feedback reports used in other research or found on the Internet, for example a bar graph, pie chart, line graph, point graph, area graph, box plot, Kaplan‐Meier graph or a funnel plot. Moreover, a distinction was made between graphs for outcome indicators such as survival and PROs and PREs, because, in general, different graphs are used for both types of data.

### 2.5 Analysis of interviews

Interviews were transcribed verbatim and qualitatively analysed using ATLAS.ti (version 7).^35^ The first two interviews of each stakeholder group were coded independently by the first and third author (LO and TV) (female, MSc, first author; male MSc, third author; both working in the same research institute). All identified items were compared and discussed until consensus was reached. The remaining interviews were coded by the first author and checked by the third author to enhance the reliability and validity of the results. The same two researchers then categorized all identified items into the interview topics. Subcategories of all codes dealing with the same subject were made by the two researchers within each category, resulting in a code tree. For example, a division into three subcategories was made within the category “Why do you prefer to receive feedback?”: individual level, hospital level and national level. Or, in the category “How do you prefer to receive feedback”, all codes regarding distribution of the report were compiled, thereby forming a subcategory. Disagreement was discussed between the two researchers and if necessary with the last author (RH) (female, PhD, last author) until consensus was reached.

## 3 RESULTS

### 3.1 Study population

For the patients as stakeholders, a response rate of 76% was reached, because three patients did not participate due to time constraints or did not respond to the e‐mail or reminder. A total of eight patients and the chairmen of both patient associations participated in the semi‐structured interviews, all in person (Table 1).

**Table 1:** Characteristics of participating patients^a^

Variable		(n=10)
Age, y	Mean	59.4
Sex, n	Female	4
	Male	6
Education level, n^a^	Medium and lower	4
	High	5
Type of tumour, n^a^	Larynx	4
	Oral cavity	5
Type of treatment, n^a^	Operation	2
	Chemoradiation	1
	Operation & radiotherapy	5
	Operation & chemoradiation	1
Year of diagnosis^a^		1997–2013

a Excluding the chairman of a patient association, who was not a patient.

The medical specialists and allied health professionals had a response rate of 94% and 69%, respectively. Reasons for not participating were time constraints, the person did not belong to the board of the national foundation for allied health professionals anymore or the person did not respond to the e‐mail or the reminder. A total of 15 medical specialists (n=15) and nine allied health professionals participated in an interview (n=9), either by telephone (n=18) or in person (n=6) (Table 2).

**Table 2:** Characteristics of participating professionals (N=24)

Variable	N
Dutch Head and Neck Society	15
Head and Neck Oncology Centres	10
Affiliated centres	5
Dutch Head and Neck Allied Health Professionals Group	9
Head and Neck Oncology Centres	7
Affiliated centres	2

The professions of these members included three radiation oncologists, two medical oncologists, five oral and maxillofacial head and neck surgeons, five otorhinolaryngologist head and neck surgeons, one speech therapist, two physiotherapists, two dieticians, two oral hygienists and two nursing consultants. Furthermore, the health insurers had a response rate of 75%, because one health insurer was not willing to participate. In total, three health insurers participated in an interview, either by telephone (n=1) or in person (n=2).

### 3.2 Preferences

**Figure 1:**
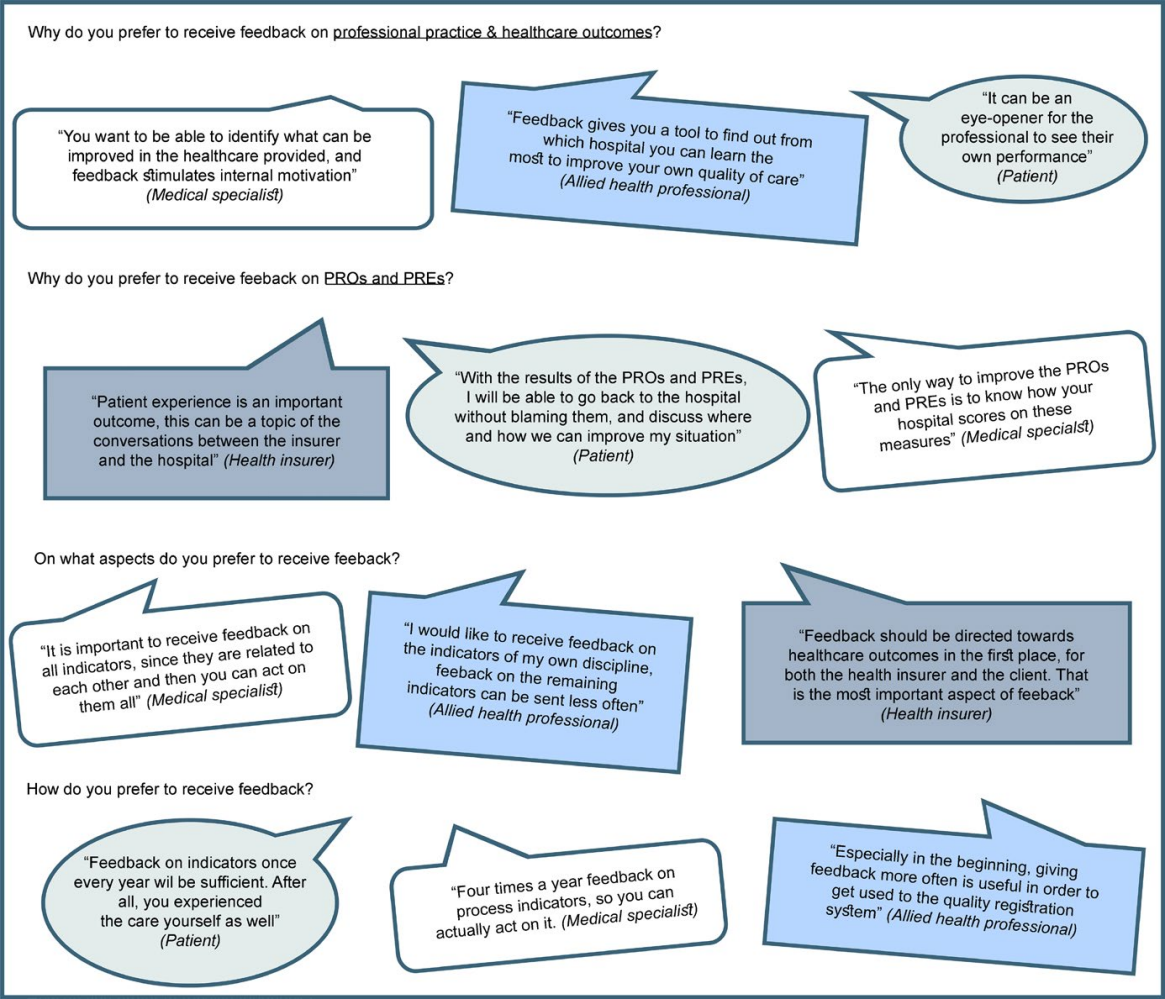
Quotes from different stakeholders on the main research questions

Tables 3, 4, 5 and 6 present an overview of the preferences of patients, professionals and health insurers regarding the three topics. In the following paragraphs, the preferences have been summarized. In addition, Figure 1 presents quotes from different stakeholders on the main research questions.

**Table 3:** Why do you prefer to receive feedback?

Subject	Patient	Medical specialist	Allied health professional	Health insurer
Feedback on indicator^a^	At an individual level—Patients: + Patients are curious + Feedback is useful for future patients + To give patients more information about the health care process + To give patients the opportunity to choose the best hospital (although some patients state that there is no option to choose, due to distance and other factors and the fact that patients prefer a treatment first) − Patients may not be interested − Feedback is not of any value to the patient − Patients might regret their decision for their treatment in that specific hospital if data become transparent/public At an individual level—Professionals: + To give doctors more insight into the health care process; an eye‐opener + A way to improve health care instead of a threat to the professional At a hospital level: + To motivate professionals to perform better + To monitor health care in hospitals At a national level: + To compare hospitals with each other and visualize the differences, although some patients consider this to be a difficult task + Important to act upon the feedback reports	At an individual level—Patients: − Feedback can result in wrong interpretations by patients − Patients are possibly not interested in indicators At an individual level—Professionals: + To become better aware of the outcomes − Feedback can result in wrong interpretations by professionals At a hospital level: + To see how other professionals in your hospital function; to keep everyone focussed + Feedback as a stimulating factor to improve performance + To know where the weak points are in your hospital + To better organize the health care process + Important to develop improvement plans: First, let the hospitals change within their hospital and improve health care + Important to put quality on the agenda in your hospital in order to pay more attention to feedback At a national level: + To compare all hospitals with each other + To increase national health care + To improve outcomes nationwide	At an individual level—Patients: + Patient can engage in the conversation with professionals if the delivered care does not meet the conditions − Feedback can result in wrong interpretations by patients At an individual level—Professionals: + To see how your colleagues are working + To create more awareness in order to deliver good health care as a professional + To pay attention to indicators, because these are easily forgotten − Feedback can result in wrong interpretations by professionals − No interest in results of indicators At a hospital level: + To see how well your hospital is functioning and from which hospital you can learn + To see which processes work in other hospitals + Feedback gives tools to engage conversations with colleagues + To put pressure on the board of directors + Important to create a structure where improvement is possible and to develop improvement plans + Put quality on the agenda in your hospital − Feedback is just a small part of health care; health care itself is about the whole figure At a national level: + To compare and to improve together + To improve or develop (new) options for treatment	At an individual level—Patients: + To represent patients’ interests + To inform patients where best care is delivered At an individual level— Professionals: + To engage in conversation between professional and health insurer At a hospital level: + To improve quality of care + To purchase by value + To engage in conversations with hospitals and to take actions if the care delivered is of inferior quality, not to punish hospitals + To measure quality of integrated health care instead of measuring quality of separate parts of the health care pathway + Put quality on the shared agenda of health care providers and health insurers At a national level: + To develop demands to improve quality of care + To compare hospitals for care procurement + To set up best practices + To ensure that hospitals do not see the health insurance company as the enemy
Feedback on PROs and PREs	At an individual level—Patients: + Patients are curious + To reflect and create awareness for the patient + To engage in the conversation with relatives, peers and professionals − Patients may not be interested − Feedback might be hard to deal with − Feedback about your own experiences and quality of life makes it less useful At an individual level—Professionals: + To create more empathy in professionals towards patients + Feedback might be more relevant and convenient for the nurse instead of the doctor − Feedback can influence the patient–professional relation At hospital level: + To improve quality of health care according PROs and PREs At national level: + To give insight into which hospital performs best on PROs and PREs	At an individual level—Patients: + Important to give all results back to the patient, also your own PROs and PREs At an individual level—Professionals: + Interesting to see results of PROs through time At a hospital level: + Use PROs and PREs for research on prognostic factors + To improve by knowing how your hospitals’ scores on PROs and PREs At a national level: + To benchmark with other hospitals	At an individual level—Patients: + To compare scores of patients on PROs and PREs At an individual level—Professionals: + It is also about “how” the patient lives instead of “if” the patient lives At a hospital level: + To improve by knowing how your hospitals scores on PROs and PREs At a national level: + To compare scores of patients on PROs and PREs within a healthy population	At an individual level—Patients: + To send patients to the best performing hospital At an individual level—Professionals: + To better know what the patient wants At a hospital level: + To use patient experiences to improve quality of care in hospitals At an national level: + PROs and PREs are part of the health care delivered

a Indicators are defined as outcome indicators, process indicators and structure indicators. Outcome indicators refer to complications, survival and recurrence rate.

**Table 4:** On what aspects do you prefer to receive feedback?

Subject	Patient	Medical specialist	Allied health professional	Health insurer
Interest in specific indicators	Interest in health care indicators that match the care received by the patient Interest in indicators that are considered to be relevant for the patient Feedback on all indicators to find out whether you missed specific care	No consensus on content of indicators: interested in all indicators on one hand, or only interested in specific outcome indicators on the other hand	Interested in indicators of allied health professionals; the remaining indicators are mainly for information (they also mentioned the relevance of receiving feedback on all indicators because they are part of one patient‐care pathway)	Mainly interested in outcome indicators. Process indicators are necessary to monitor the processes that underlie the outcome indicators

**Table 5:** How do you prefer to receive feedback?

Subject	Patient	Medical specialist	Allied health professional	Health insurer^a^
Frequency and timing	General: Do not give feedback on PROs and PREs too often Frequency: Indicators: once a year PROs/PREs: once a year Timing: Either before treatment or after the diagnostic phase (there is more stress during the diagnostic phase) When the indicators are relevant in the health care process	General: Preference for receiving more feedback at the beginning Preference for receiving feedback more often when severe deviations in the data appear Frequency: Process indicators: 1–4 times a year (depending on the possibility of improving in the meantime) Outcome indicators: 1–2 times a year	General: In the beginning, feedback could be given more often Frequency: Process indicators: 1–2 times a year Outcome indicators: 2–4 times a year	
Report method	General: Figures with an explanation of the content and “how to read” Dosing of the amount of information in smaller parts Keep the target audience in mind (eg, colour blind, use of medical terms, level of degree) Use of average scores: Give feedback with average national scores on the PROs and PREs, but be aware of consequences:(Positive: give insight into where you stand, give a boost and lean on results of other patients)Negative: insecure or discouraging feelings National average scores on indicators of more interest for patient organizations and professionals Distribution of feedback: Feedback by e‐mail or a patient portal A conference is a good idea for paying more attention to head and neck cancer	General: Find a balance between giving feedback and giving too much information Give an overview of the results first, followed by the details Present it in such a way that one can easily understand without explanation Use of average scores: Give feedback on own scores compared with the average score, the best hospital and the worst hospital when data will be presented anonymously Give the scores of all hospitals including national average scores, the best and the worst performing hospital Distribution of feedback: Feedback by e‐mail First, the hospitals can try to work it out on their own, then they can ask for more background information or explanation of the investigator Organize a committee to monitor the content and format of the feedback report Take case mix into account Give feedback on the quality of data Use specific themes each year when data will be compared on a national level National feedback in the form of a conference is a useful idea; however, feedback in your own organization will be useful as well	General: Keep it simple Give an overview of own indicators first, followed by the remaining indicators Use of average scores: Give feedback with the scores of each hospital; use of average scores depends on the goal of the feedback Give feedback on own scores compared with the national average scores to see how your hospital is functioning, because one prefers not to be presented as a “bad” hospital Distribution of feedback: Feedback by e‐mail A meeting in the hospital organized by the investigator is preferred for more background information and explanation of the results National feedback in the form of a conference is a useful idea; however, it is better to discuss feedback in your own hospital first	
Transparency	General: + Transparent for patients − Be careful that feedback is not interpreted carelessly − Be aware that results can change in a short time span Method: Ask permission of the patient to receive their own results or the results of the general population Make sure that you can trust the data: if a doctor gathers the data they could be less reliable	General: + The only way to improve is to make data public/transparent + To feel a sense of responsibility towards the population − Be careful with transparency; it is about vulnerable data Method: Set up a committee to decide on issues related to transparency Be critical in what a patient is able to understand Make sure the specific hospital cannot be derived from the data presented Only give feedback using scores of all hospitals when data will be presented anonymously Investigate whether there are specific conditions to make the data public. Make sure data are correct	General: + Being transparent is good + The only way to improve is to make data public/transparent − You cannot influence the indicators Method: No anonymous feedback, only in the start‐up phase Be critical in what a patient can understand Make sure that professionals are able to influence the indicators	General: + To feel a sense of responsibility towards the population + Visualize to improve health care Method: Visualize as transparently as possible what type of care is delivered

a There is no information available on how the health insurers prefer to receive feedback because they prefer to receive raw data to develop their own figures.

**Table 6:** Preferences on the various figures^a^

Figures	General perspective	Patient	Medical specialist	Allied health professional
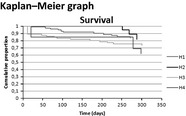	+ Gives a clear overview + A classical way of presenting data, often used in science + A good way of presenting, mainly for outcome indicators + Most useful when there are big differences − Might be difficult for patients (and for some professionals too) to interpret	− For outcome indicators: too difficult to understand	+ For outcome indicators: gives a clear overview, seen as the classical way to present outcomes Preference for this figure and a bar graph to present outcome indicators	− For outcome indicators: too difficult to read
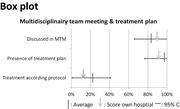	− Gives a clear overview at a glance − A clear overview of how your hospital scores compared with the rest − Difficult to read for patients, and for some professionals as well	− For outcome indicators and process indicators: too difficult to understand	+ For outcome indicators and process indicators: gives a clear overview at a glance	+/− For outcome indicators: for some people it could give a very clear overview, for others it is difficult to read − For process indicators: gives a less clear overview and is more difficult to interpret
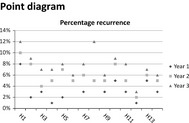	+ Does not give a clear overview whether all information is added into the same figure; + Visualize all the information you want		+ For outcome indicators: gives an unclear overview	+ For outcome indicators: gives an unclear overview
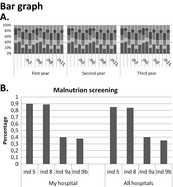	+ For a clear presentation of data + For patients, the bar graph is visually attractive and more clear to see compared with a pie chart, especially for elderly people +/‐ Can be used for the first overview, but afterwards you would prefer more detail +/‐ Insightful, although it might be difficult to read if more categories are used in one chart − Can become a very muddled and unclear figure	+ For outcome indicators (A): gives a clear overview + For PROs and PREs: gives a more clear overview − For process indicators (B): too difficult to understand Preferences for this figure and a pie chart to present PROs and PREs	+ For process indicators (B): insightful + For PROs and PREs: is easier to read compared with a pie chart +/− For outcome indicators (A): can be difficult to read when several categories are used in the outcome indicators Preferences for this figure to present outcome indicators and process indicators. A Kaplan‐Meier graph is also preferred for outcomes	+ For process indicators (B): gives a clear overview +/− For outcome indicators (A): can be difficult to read when several categories are used Preferences for this figure to present outcome indicators and process indicators Not a specific preference for a pie chart or a bar graph to present PROs and PREs
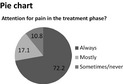	+ Gives a clear overview, especially when there are big differences + Mainly for younger patients	+ For PROs and PREs: gives a more clear overview Preferences for this figure and a bar graph to present PROs and PREs	+ For PROs and PREs: gives a clear overview Slight preferences for this figure to present PROs and PREs compared with a bar chart	+ For PROs and PREs: gives a more clear overview and is easier to read Not a specific preference for a pie chart or a bar graph to present PROs and PREs
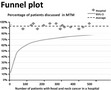	+/− Gives a good overview but also contains a lot of information − Can be a complicated and unclear figure	− For process indicators: too difficult to understand	+ For process indicators: gives a clear overview Difficult to read at a glance	− For process indicators: gives a less clear overview and is more difficult to interpret
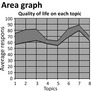	+ Advantage is that all information is in one figure − Not clear; the figure will probably be easier to understand with an explanation − Difficult figure to understand directly − Difficult for a patient to read; they never see this figure in daily life	− For PROs and PREs: too difficult to understand	+/− For PROs and PREs: more clear when a explanation is given, although it remains difficult as well: patients have probably never seen area graphs before	+/− For PROs and PREs: more clear when an explanation is given, at a glance it is a difficult figure to understand
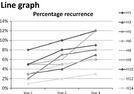	+ Both insightful and unclear		+/− For outcome indicators: it gives a lot of information but it is also confusing	+/− For outcome indicators: it gives a large amount of information but it is also confusing

a There is no information available on how the health insurers prefer to receive feedback because they prefer to receive raw data to develop their own figures.

### 3.3 Why do you prefer to receive feedback?

#### 3.3.1 Feedback on professional practice & health care outcomes

In general, all stakeholders prefer feedback on professional practice and health care outcomes (Table 3). The main reason mentioned was that feedback can improve health care, either at an individual level, hospital level or national level. Feedback can create awareness. It can also be a method for reflection on yourself and on peers or colleagues.

Feedback can also be used to benchmark and improve health care with all health care providers together. Stakeholders agree that it is important to act upon feedback, either by developing improvement plans or by putting the feedback on the agenda as a start. Both actions will result in more attention to the use of feedback in the hospital. Additionally, feedback can engage quality of care discussions among and with professionals, patients and health insurers about the care delivered and the experiences of all parties involved. All four stakeholders agree that patients might not be interested in or might not understand the feedback on professional practice. In addition, health care professionals themselves mentioned that not all health care professionals would be able to understand the feedback properly.

Health insurers specifically stated that it is not their aim to judge hospitals for the good work they deliver, but to apply feedback as a discussion tool in their interactions with care providers. Health insurers consider feedback to be a necessary tool to improve care for the patient (eg, by informing the patient and representing their interest based on the feedback). In comparison, professionals consider feedback to be a method to improve care together with the patient.

#### 3.3.2 Feedback on PROs and PREs

The main reason for patients to want to receive feedback on PROs and PREs is to be able to engage in the discussions with peers or professionals regarding their quality of life, experiences and received care.

Medical specialists see the PROs and PREs as another way of benchmarking and improving health care. Allied health professionals mention that feedback on PROs and PREs are of particular interest, because they are about “how” the patient lives instead of “whether” the patient lives for a longer period. For health insurers, PROs and PREs form a part of the outcome indicators and are necessary to measure quality; patient experiences are necessary to improve health care.

### 3.4 On what aspects do you prefer to receive feedback?

Patients would prefer to receive feedback on the professional practice that matches their health care pathway; for example, the patient does not want to receive feedback on the professional practice of the physiotherapist if the patient did not receive any physiotherapy at all (Table 4). Medical specialists and health insurers alike mention that health care outcomes are most relevant when they can be compared with the aspects of professional practice, because they deal with the effect of the treatment.

In contrast with medical specialists, allied health professionals mention more frequently that they are more interested in feedback on the professional practice of their own discipline. However, both groups agree that feedback on all health outcomes and aspects of professional practice is needed, because they also form part of the health care pathway of the patient.

### 3.5 How do you prefer to receive feedback?

#### 3.5.1 Frequency and timing

Patients prefer to receive feedback when the specific health outcomes and aspects of professional practice have become relevant in their disease process. They prefer to receive this feedback by e‐mail or through a patient portal. In terms of frequency, patients mentioned that, for all indicators (including PROs and PREs), feedback once a year would be sufficient (Table 5). Patients would prefer to receive feedback for the first time after the diagnostic phase, because then their stress level will be lower compared with during the diagnostic phase.

Both medical specialists and allied health professionals agree that feedback should be given more often in the start‐up phase of a quality registration. In this way, users will get used to receiving feedback and will act on it.

Medical specialists and allied health professionals differ on the frequency of feedback: medical specialists prefer to receive feedback on process indicators (1‐4 times a year) more often compared with outcome indicators (1‐2 times a year). However, for allied health professionals, this is exactly the opposite.

#### 3.5.2 Report form

Patients mentioned that feedback should be well balanced and an explanation of the figure or graph should be given. Furthermore, patients mention that average scores of how all hospitals perform on professional practice might be of more interest for professionals and patient associations. With regard to average scores of PROs and PREs, patients mention that it gives them an insight into where they stand, as well as possibly giving a boost. On the other hand, information about the average quality of life of other patients might result in insecure or discouraging feelings of patients regarding their own care status.

Professionals agree that the report should be simple as well as giving an overview of the indicators, followed by more in‐depth information. In addition, they are all in doubt about displaying average scores or specific scores of hospitals in public. They fear that it could result in reputational damage when the hospital is pictured as a lesser performing hospital. Professionals agree that feedback should preferably be given by e‐mail.

In contrast to medical specialists, allied health professionals prefer to receive the indicators of their own discipline first, followed by the remaining indicators. In addition, allied health professionals would prefer a meeting around the feedback with more background information. Medical specialists prefer to discuss feedback within their hospital before asking for more background information.

Furthermore, prior to giving feedback on PROs and PREs to patients, medical specialists feel that professionals should question the preferences of the patient regarding receiving their own results or the results of the general population. Professionals should also ask patients whether results on PROs and PREs might be consulted by professionals.

#### 3.5.3 Transparency

Patients and professionals alike are cautious about transparency of data. They are worried about the quality of data and the risk of misinterpretation. Medical specialists suggest organizing a committee to decide on issues concerning transparency. In contrast, allied health professionals are in favour of making data public and have less stringent requirements for making data public compared with medical specialists. Health insurers mention that they feel a duty to take responsibility to the population. In order to improve care, it is important to visualize delivered care.

#### 3.5.4 Type of graph for feedback on indicators

Patients mentioned that feedback figures for professional practice are difficult to read for patients in general (Table 6). In contrast, figures for health outcomes are easier to read for patients. Professionals also confirm that patients might not be able to read the feedback on health outcomes and professional practice.

For both health outcomes and professional practice, patients as well as professionals prefer bar graphs because they are easy to read. Other preferred graphs for medical specialists are Kaplan‐Meier graphs and box plots for survival indicators and process indicators, respectively. Allied health professionals mention that box plots, Kaplan‐Meier graphs and funnel plots give a less clear overview and are more difficult to interpret.

#### 3.5.5 Type of graph for feedback on PROs and PREs

Patients mention that figures for this kind of feedback are easier to read compared with figures for process and structure indicators (Table 6). Patients prefer both a pie chart and a bar graph. In general, patients prefer a figure over plain text. Professionals have a slight preference for a pie chart compared with a bar graph.

## 4 DISCUSSION

This exploratory study investigated the preferences of receiving feedback on outcome, process and structure indicators in the DHNA from four different stakeholder perspectives: patients, medical specialists, allied health professionals including nurses and health insurers. It shows that stakeholders agree that use of feedback can improve health care by creating awareness, by enabling reflection on oneself and colleagues, by benchmarking to others and by engaging quality of care discussions between parties involved. Patients prefer to receive feedback on quality indicators that match their health care pathway, whereas medical specialists and health insurers are interested mainly in outcome indicators. Furthermore, all stakeholders prefer a bar graph for feedback on most health outcomes and professional practice. In addition, patients prefer a pie chart for PRO experiences, while a Kaplan‐Meier graph is preferred specifically for survival curves by medical specialists. Feedback should be simple and intended to give an overview firstly. Moreover, it should be sent by e‐mail with a frequency of 1‐4 times a year.

Other literature is focussed mainly on preferences of patients or clinicians, but this study includes preferences of four different stakeholders.^22^, ^36^, ^37^ It is also directed towards different types of indicators, namely process, structure and outcome indicators. Furthermore, it gives a clear overview of why, what and how patients, professionals and health insurers prefer to receive feedback.

Our study confirms that feedback is a method for reflection and for creating awareness, resulting in a change in behaviour.^1^, ^22^ Also, patients and professionals mention that knowing the hospital's scores on PROs and PREs can improve the quality of care. Greenhalgh showed already that the use of PROs in clinical practice is valuable in improving the discussion and detection of health‐related quality of life problems.^38^

In line with previous literature and irrespective of the stakeholder, simple formats, such as bar graphs, were generally preferred to more complex graphical information.^20^, ^39^, ^40^ Regarding PROs and PREs, our study shows that both a pie chart and a bar graph are preferred by patients. Professionals have a slight preference for a pie chart over a bar graph. Hildon et al.^41^ described that patients often prefer a bar graph, because it is a clearer graph visually. Moreover, patients’ preferences for a bar graph are in line with Kuijpers et al.^20^ In addition, Hildon et al. described that a funnel plot was difficult to read for patients, which our study confirms as well.^41^

Although our patient population prefers a figure over plain text, they would also prefer an explanation to go with the figure. This is in line with Brundage et al., who stated that patients did not wish to receive HRQL information out of context or without explanation,^39^ and also with Tufte, who gave an overview of the characteristics that a well‐readable graph should have.^42^

### 4.1 Limitations

The fact that only three health insurers participated in the study could be considered a limitation. This is probably too small to reach saturation (the point at which no new information was mentioned in the interviews).^34^ However, the health insurers shared the same thoughts on the topics discussed. Bias may have occurred when selecting the patients, because it is possible that patients with a higher social status and adequate communication skills were selected by each hospital, resulting in a less representative patient population. HNC is associated with poor socio‐economic circumstances.^43^ In the interviews, it became clear that it was difficult for patients to understand the feedback regarding health outcomes, such as recurrence rates. Therefore, the interviews with patients were directed mainly towards the use of feedback on PROs and PREs, when we spoke about “health outcomes”. Questions for the health insurers merely focussed on the goal of feedback, because the insurers mentioned that they prefer raw data instead of receiving a complete report.

Lastly, there might be an overvalue of positive preferences for feedback. This study shows that all stakeholders are positive about receiving feedback on professional practice and health care outcomes. However, if this view would manifest itself in action, you would expect that the literature on implementation of audit and feedback would show much larger and more consistent effect sizes. This is similar to the situation in which adherence to clinical guidelines is still low and clinicians often overstate their adherence to the guidelines.^44^, ^45^, ^46^ Knowing how stakeholders prefer to receive audit and feedback does not assure that they will actually respond to it. Therefore, it is necessary to test the response in practice.

## 5 CONCLUSION

This exploratory study shows that preferences for receiving feedback between patients, professionals and health insurers differ regarding content but not regarding layout. Therefore, reports tailored to these preferences are recommended. Using this information, the effect of audit and feedback can be improved by adapting the feedback format and contents to preferences of stakeholders. As a result, this could potentially improve quality of care. A next step is to test in practice to what extent professionals actually respond if audit and feedback suit their preferences.

## COMPETING INTERESTS

The authors declare that they have no competing interests.
